# Tele-delivered caregiver coaching for autism in South Africa – A mixed-methods study of acceptability, appropriateness and feasibility

**DOI:** 10.1177/20552076261459555

**Published:** 2026-06-11

**Authors:** Marisa Viljoen, Zahrah Ismail Dawood, Noleen Seris, Nokuthula Shabalala, Minkateko Ndlovu, Petrus J de Vries, Lauren Franz

**Affiliations:** 1 Centre for Autism Research in Africa (CARA), Division of Child & Adolescent Psychiatry, 37716 University of Cape Town, Cape Town, South Africa; 2 Division of Communication Sciences and Disorders, Department of Health and Rehabilitation Sciences, 37716 University of Cape Town, Cape Town, South Africa; 3 Duke Center for Autism and Brain Development, Department of Psychiatry and Behavioral Sciences, Duke University, Durham, North Carolina, USA; 4 Duke Global Health Institute, 199688 Duke University, Durham, North Carolina, USA

**Keywords:** telehealth, autism, LMIC, acceptability, appropriateness, feasibility, barriers, facilitators, digital divide

## Abstract

**Objective:**

Tele-delivered interventions can benefit autistic children and families in low-resource settings, but the digital divide may worsen existing disparities. To address this, we adapted a Naturalistic Developmental Behavioural Intervention (NDBI)-based caregiver coaching program for tele-delivery to enhance fit for low-resource families in Cape Town, South Africa. This manuscript examines the contextual fit (acceptability, appropriateness, feasibility) and implementation facilitators/barriers of the adapted intervention.

**Methods:**

A mixed-method design was used to assess implementation outcomes in a pilot study of 16 participants (caregivers and practitioners), with quantitative (Acceptability of Intervention Measure, AIM; Intervention Appropriateness Measure, IAM; Feasibility of Intervention Measure, FIM) and qualitative data. Quantitative data were analysed using descriptive statistics and qualitative interview data using framework analysis.

**Results:**

Quantitative data showed high scores for acceptability, appropriateness and feasibility (AIM intervention materials = 4.5/5; AIM session structure = 4.4/5; IAM intervention materials = 4.6/5; IAM session structure = 4.5/5; FIM session structure = 4.4/5), supported by qualitative findings. Barriers included small text size on intervention materials and internet connectivity. Facilitators included minimal text in intervention materials, an accessible delivery platform (WhatsApp), collaborative caregiver-practitioner relationships, data and technical support, protected practitioner time, and school leadership support.

**Conclusion:**

This is one of the first studies in a LMIC to assess implementation of an asynchronous autism caregiver coaching program delivered entirely via a low-cost instant messaging platform (WhatsApp). Findings suggest the tele-delivered intervention fit well in this low-resource South African setting. Results are promising, and evaluating caregiver and child outcomes is a key next step.

## Introduction

There is growing recognition of the need to provide accessible supports and services for autistic individuals across the lifespan.^1,2^ Early intervention is especially important, as it promotes development during a critical period when the brain is highly receptive to learning.^3,4^ Naturalistic Developmental Behavioural Interventions (NDBI) are a group of evidence-based early interventions that support child development.^2,5,6^ They are delivered in natural, everyday activities such as mealtimes, getting dressed or playing with toys. NDBI combine developmental strategies (e.g. following the child’s lead and modelling gestures and language) and behavioural strategies (e.g. prompting skills and responding to the child’s request by giving them what they ask for within the interaction). The Early Start Denver Model (ESDM),^7^ is an NDBI that can be delivered through caregiver coaching, where caregivers are taught to use these strategies with their children during everyday activities.^6,8^ Despite its promise, early intervention remains out of reach for most autistic children in low- and middle-income countries (LMIC), where over 90% are unidentified and lack access to services.^2,9–11^ This service gap is driven by factors including a shortage of trained professionals, limited availability of services, poor transportation infrastructure, and poverty – leaving many families forced to choose between meeting basic needs and accessing therapy and support for their autistic family member.^2,12–16^

Tele-delivered interventions offer a potential solution to these challenges by enabling remote access to services, and reducing travel time and costs.^17–20^ In high-income countries (HIC) tele-delivered caregiver coaching as an early autism intervention approach has been shown to be feasible and effective in improving child communication and language skills.^19–27^ However, these findings cannot be assumed to apply to LMIC, where contextual factors including technological infrastructure, family characteristics and service delivery systems differ significantly. One key factor to consider in LMIC is the digital divide – which refers to the gap in access to digital devices and reliable internet, often due to differences in income, location, education, or available infrastructure.^18,28^

To our knowledge, only two LMIC-based studies, in China^29^ and India,^30^ have evaluated tele-delivered caregiver coaching as an early autism intervention approach. While both studies showed the approach was acceptable, appropriate, and feasible, technical issues with the video conferencing platforms were reported, including unstable internet connectivity affecting the ability to run Zoom sessions as well as poor video and audio quality of Zoom calls.^29^ Interestingly, studies of tele-delivered caregiver coaching both in LMIC^29–31^ and HIC,^21^ have used video conferencing platforms like Zoom,^32–35^ which require stable internet and high-performance devices with fast processors and quality cameras to support synchronous (real-time) or a combination of synchronous and asynchronous (store-and-forward or self-paced) intervention delivery. As a result of the digital divide, most families in LMIC settings own low-cost digital devices and have limited access to reliable, high speed internet. Many rely on low-cost smartphones with basic features like calling, texting, and internet browsing. Free apps that use little data such as WhatsApp (an app that allows users to send text messages, voice messages, photos, videos, and make voice and video calls over the internet) are often used on these devices for messaging and calling.^36–39^ This highlights the importance of designing telehealth programmes that fit the digital context,^2,18,40^ so that coaching interventions do not widen existing service access gaps. In practice, this requires a clear understanding of which digital platforms (e.g. Zoom, WhatsApp) and session structure (e.g. synchronous or asynchronous) work best for families in a specific setting. Examining implementation outcomes of a tele-delivered intervention, including acceptability, appropriateness and feasibility can help us understand what works, what doesn’t, and why (see table 1 for definitions).^41^ Furthermore, exploring implementation outcomes can delineate barriers that make implementation more challenging and facilitators that aid in implementation, in order to inform contextual adaptations of the tele-delivered intervention to improve fit.

**Table 1.** Definitions of implementation constructs as defined by Proctor, 2011.^41^

Implementation construct	Definition
Acceptability	The perception among stakeholders that the approach is agreeable, palatable, and satisfactory
Appropriateness	The perceived fit, relevance, or compatibility of the approach for a given setting, provider, or consumer
Feasibility	The extent to which the approach can be successfully used within the given setting

### Context and aims of the study

**Figure 1.**
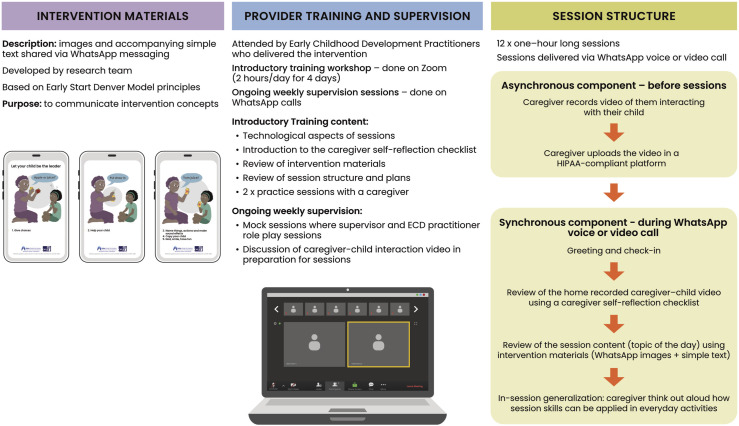
The three technology components of tele-delivery: 1) intervention materials, 2) provider training and supervision, and 3) session structure.

In Cape Town, South Africa, the COVID-19 pandemic required that an in-person NDBI-informed caregiver coaching intervention based on the principles of the Early Start Denver Model (ESDM) be adapted rapidly for tele-delivery.^42–44^ Originally delivered in-person to families from low-resource settings by non-specialist Early Childhood Development (ECD) practitioners, the shift to remote delivery created an opportunity to evaluate the contextual fit of the adapted approach. Viljoen et al.^44^ identified the adaptations made to transition the in-person intervention to tele-delivery and explained the reasons for each. These were grouped into three main technological components: 1) materials used to communicate intervention concepts to caregivers, 2) provider training and supervision, and 3) session structure (see Figure 1).

As seen in Figure 1 the first technological component, intervention materials, were comprised of WhatsApp images with accompanying simple text to communicate intervention concepts across the 12 coaching sessions. Intervention materials were developed by the research team based on ESDM principles. The second technological component, provider training and supervision, consisted of a short introductory training over Zoom (two hours per day for four days) that was attended by ECD practitioners who delivered the intervention. ECD practitioners had prior training in the intervention content as they had been conducting in-person caregiver coaching prior to the COVID-19 pandemic.^42.43^ As a result, training focused on technological aspects and session structure, with only a brief overview of the intervention materials (see Figure 1). Once caregiver coaching sessions began, weekly online supervision was provided via WhatsApp calls. The third technological component, session structure, featured a hybrid format that combined asynchronous and synchronous elements. For the asynchronous element caregivers recorded and uploaded a 5-minute long video of them practicing the session skill with their child. Since autistic children were not present during the WhatsApp session calls the videos acted as a bridge between coaching sessions and real-life, allowing caregivers to show how they applied intervention strategies at home. The synchronous part comprised of coaching sessions that were conducted via end-to-end encrypted WhatsApp voice or video calls. Coaching sessions had a set structure. They began with greeting and check-in, followed by a review of the caregiver’s home-recorded video using a self-reflection checklist with questions reflecting on caregiver use of skills. Next, the coach and caregiver went over the session topic (the ‘topic of the day’) using the intervention materials (WhatsApp images with simple text). Finally, during the generalization phase, the caregiver was supported to think out loud about how to use the session skill in everyday activities. Caregiver and coaching fidelity were monitored throughout the intervention.

The aim of this manuscript was to evaluate the acceptability, appropriateness and feasibility of the three key technological components of the tele-adapted intervention (intervention materials, provider training and supervision and session structure) from the perspectives of various stakeholders involved in intervention delivery. Where applicable, we also identified implementation barriers and facilitators. Intervention effectiveness will be evaluated in a separate manuscript.

## Methods

### Study design

A mixed-method convergent design^45^ was used in this pilot study to evaluate implementation outcomes (acceptability, appropriateness and feasibility) of the adapted tele-delivered intervention and to identify implementation barriers and facilitators. Quantitative and qualitative data were collected concurrently and analysed in parallel. Results were then integrated through data merging with joint display.^45–47^ Quantitative data were collected on the acceptability and appropriateness of intervention materials, as well as on the acceptability, appropriateness, and feasibility of intervention session structure. Qualitative data were collected on the acceptability, appropriateness, feasibility of intervention materials, provider training and supervision and session structure, as well as implementation barriers and facilitators.

### Participants

All individuals who participated in the tele-delivered intervention, ECD practitioner training and supervision were invited to participate. Participants included three South African certified ESDM therapists who supervised intervention delivery, two non-specialist ECD practitioners who delivered the coaching program, one school supervisor who provided logistical support to ECD practitioners, and ten caregivers of young autistic children who participated in the telehealth coaching sessions.

Inclusion criteria for session supervisors were certification as an ESDM therapist and participation in training and supervision of ECD practitioners. For ECD practitioner and school supervisors, inclusion criteria included employment by the Western Cape Education Department and involvement in delivery of telehealth caregiver coaching sessions. Inclusion criteria for caregiver-child dyads were (1) the child was between 18 and 72 months of age, (2) the subject’s family spoke isiXhosa, isiZulu, Afrikaans, or English, (3) the child’s ethnicity/race was African, Coloured (a South African term for mixed race) or Indian, (4) the child lived within an area served by the recruitment sites, (5) the child met criteria for an ASD diagnosis based upon DSM-5 and informed by the ADOS-2 administered by research reliable raters, and (6) the caregiver was ≥ 18 years old. Exclusion criteria for caregiver-child dyads were (1) the child had a neurological disorder of known aetiology (e.g., Fragile X), (2) a caregiver-child dyad that was unable to attend assessments and 12 coaching sessions, (3) the child had significant sensory or motor impairment, (4) the child had major physical abnormalities, and (5) the child had a history of serious head injury and/or neurological disease.

### Procedures

All procedures were administered remotely. Prospective participants were invited to complete enrolment and consent telephonically, following consent procedures approved by the University of Cape Town Human Research Ethics Committee (301/2015 & 468/2019) and the Duke Health Institutional Review Board (Pr00103045). Quantitative and qualitative data were collected telephonically. Quantitative data from caregivers was collected during post-intervention assessments, while quantitative data from session supervisors, ECD practitioners, and school supervisors was gathered after they had facilitated or supervised all twelve sessions for at least one caregiver–child dyad. Caregiver qualitative in-depth individual interviews occurred after completion of at least ten (of twelve) caregiver-coaching sessions, to allow for participant familiarity with the session structure and intervention materials. Session supervisor, ECD practitioner and school supervisor qualitative interviews were conducted after facilitating or supervising twelve (of twelve) sessions for at least one caregiver-child dyad. The project coordinator and a research assistant, neither of whom had participated in the coaching sessions, conducted interviews. Interviews were audio-recorded with participant consent and transcribed verbatim. Data collection occurred in Cape Town, South Africa between September 2020 and July 2021.

### Ethics and protocol registration

The study was a collaboration between the Centre for Autism Research in Africa at the University of Cape Town (UCT) and the Duke Centre for Autism and Brain Development at Duke University. Data collection methods were therefore approved by UCT Human Research Ethics Committee (301/2015 & 468/2019) and Duke University Health System Institutional Review Board (Pr00103045). Further permissions were obtained to conduct research at Red Cross War Memorial Children’s Hospital (RCC_185) and the Western Cape Department of Education (Insert Refs). The study protocol was registered on Clinical Trials.gov (NCT04068688).

### Measures

#### Sociodemographic characteristics

At baseline, all participants provided sociodemographic information. Caregivers reported their age, relationship to the child, level of education, employment status, household income, and their child’s age, gender, home language, and caregiver-declared race. ECD practitioners, school supervisors and session supervisors reported their gender, race, level of education, and languages spoken (see supplemental material).

#### Technology access

Caregivers reported on their access to technology, including the type of phone they owned, whether they had internet access, if they used mobile data or Wi-Fi for internet access, and where they were typically located when they accessed the internet. Caregivers also rated their own technology skills on a 10-point Likert scale from 1 (very poor) to 10 (excellent) (see supplemental material).

#### Acceptability of Intervention Measure (AIM), Intervention Appropriateness Measure (IAM), and Feasibility of Intervention Measure (FIM)

The AIM, IAM and FIM are pragmatic measures that each include a 4-item questionnaire on the acceptability, appropriateness and feasibility of an intervention within a specified setting.^48^ These instruments are publicly available for research use and were applied in accordance with the authors’ guidelines, with appropriate citation of the original source; no additional permissions were required. The wording of the AIM, IAM and FIM can be customized to fit the intervention and context while maintaining the core constructs. For this study the wording was tailored to evaluate the intervention materials (referred to as ‘WhatsApp pictures’ by participants) and session structure (referred to as ‘phone sessions’ by participants) (see supplemental material). Items were rated on a 5-point Likert scale ranging from completely disagree (rating of 1) to completely agree (rating of 5).^48^ Higher scores indicate greater acceptability, appropriateness, and feasibility. Examples of items tailored for this study were “I like the phone coaching sessions” (AIM), “the WhatsApp pictures seem suitable” (IAM), and “the phone coaching sessions seem easy to use” (FIM). Psychometric properties of these measures showed high reliability within each scale both in HIC (Cronbach alpha coefficients AIM = 0.85, IAM = 0.91, FIM = 0.89) and in a LMIC context (Cronbach alpha coefficients AIM = 0.89, IAM = 0.91, FIM = 0.93).^41^

#### In-depth individual interviews

Interview guides were adapted from Curran and colleagues’ multi-stakeholder qualitative evaluation process,^49^ and informed by definitions of by acceptability, appropriateness and feasibility as defined by Proctor et al. 2011.^41^ Questions focused on three key technological components of tele-delivery, including intervention materials, provider training and supervision and session structure, in addition to implementation barriers and facilitators across these technological components. A similar interview guide, was previously used by Makombe and colleagues^50^ in the South African context. Interview guide questions varied slightly based on the participant group (session supervisors, ECD practitioners, school supervisors or caregivers). The interview guide is available from the authors upon request.

### Data analysis

#### Quantitative analysis

Descriptive statistics were used to analyse sociodemographic characteristics (n, percentage for categorical variables and range), the technology survey (n, percentage for categorical variables and range) and AIM, IAM, FIM measures (mean and range).

#### Qualitative analysis

Transcribed interview data were analysed using framework analysis, a thematic analysis method where a structured analytical codebook was used to systematically organize qualitative data.^51,52^ The analysis was guided by a combined deductive-inductive framework analysis described by Gale et al.^52^ The deductive framework was informed by the three technological components of tele-delivery (materials used to communicate intervention concepts to caregivers, provider training and supervision, and session structure) and the implementation constructs of acceptability, appropriateness and feasibility. Inductive coding was used to capture novel themes (for example, supportive caregiver-practitioner relationships) emerging from the data not pre-determined in the deductive framework (52). This is consistent with Gale et al.^52^ who describe the framework method as allowing both deductive and inductive coding. Codes in the structured analytical codebook were developed deductively, with codes derived from the research questions, and inductively, with codes emerging from the data.^51–53^ Nvivo 14 software was used to organize, code and analyse transcribed interviews.^54^ During the framework analysis two coders familiarized themselves with the data by reading and re-reading transcripts. Next, both coders read a sample of four interview transcripts that represented the full range of participants. A combination of inductive and deductive codes were assigned. Deductive codes were informed by the three technological components of tele-delivery and the implementation constructs of acceptability, appropriateness and feasibility.^44^ Inductive codes emerged based on participant perspectives not captured by deductive codes. Coders met regularly to compare and discuss codes. Through this iterative process a final codebook was generated that was used to code the qualitative dataset. Finally, each coder independently reviewed the assigned codes across transcripts, and met on a regular basis as coding progressed to check these classifications. When coding discrepancies emerged, these were discussed with the study principal investigators. No new themes emerged during coding, indicating that data saturation was reached.

#### Mixed-methods integration

Quantitative results from the AIM, IAM and FIM and qualitative results from individual interviews were integrated through data merging with joint display in a table which allows for side-by-side comparison to determine if quantitative and qualitative data offered similar conclusions (i.e., confirmation of findings), expanded insights into the results, or provided contradictory findings (i.e., discordance).^45,55,56^

## Results

### Participant sociodemographic and technology characteristics

A total of 16 participants were included in the study. Caregiver, ECD practitioner and school supervisor, and session supervisor characteristics are outlined in table 2. The group of ECD practitioners, school supervisors, and session supervisors (n=6) included three Coloured (an official demographic category in South Africa for people of mixed ancestry), one Black African, and two Caucasian females. All ECD practitioners held post-Grade 12 certificates, while session and school supervisors had completed tertiary education. All spoke English and Afrikaans, with one of the session supervisors also speaking isiZulu, isiXhosa and seSotho.

**Table 2.** Participant sociodemographic and technology characteristics.

ECD practitioner, school & session supervisor characteristics		N (%)	Range
Gender	Female	6 (100%)	-
Ethnicity/Race	Coloured*	3 (50%)	-
	Black African	1 (17%)	-
	Caucasian	2 (33%)	-
Education	Diploma/certificate	2 (33%)	-
	College/university	4 (67%)	-
Languages spoken	English	6 (100%)	-
	Afrikaans	6 (100%)	-
	isiZulu	1 (17%)	-
	isiXhosa	1 (17%)	-
	seSotho	1 (17%)	-
Caregiver Characteristics		N (%)	Range
Caregiver age in years, Mean	​	36.1	29-48
Primary Caregiver	Mother	8 (80)	-
	Father	2 (20)	-
Number of siblings living in the home	No siblings	6 (60)	-
	1 sibling	1 (10)	-
	2 siblings	2 (20)	-
	3 siblings	1 (10)	
Caregiver Education	Grade 12 (completed high school)	5 (50)	-
	Post-Grade 12 Diploma/Certificate	2 (20)	-
	Tertiary	3 (30)	-
Marital Status	Married	7 (70)	-
	Live-in Partner	1 (10)	-
	Single	1 (10)	-
	Partner does not live-in	1 (10)	-
Employment Status	Not working	5 (50)	-
	Working Part-time	2 (20)	-
	Working Full-time	3 (30)	-
Household Income/month	Less than R4,501 (∼<$244)	2 (20)	-
	R4,501-R12500 (∼>$244-$678)	2 (20)	-
	R12,501-R30,000 (∼>$678-$1628)	4 (40)	-
	More than R30,000 (∼>$1628)	2 (20)	-
Perceived economic security	Struggling	4 (40)	-
	Just getting by	2 (20)	-
	Doing OK	4 (40)	-
Mobile phone	Smartphone	9 (100)	-
Type of phone	Android	6 (75)	​
	Apple	2 (25)	​
Personal email address	Yes	9 (100)	-
Where participants connect to the internet	In the community	1 (11)	-
	Work	5 (56)	-
	Home	8 (89)	-
How participants connect to the internet	Own modem	6 (67)	-
	Free community WiFi	1 (11)	-
	Mobile phone data	6 (67)	-
Self-rated skill using the internet (scale of 1-10; 1 = very poor and 10 = excellent), Mean (SD)	​	8.1	5-9

ECD = early childhood development.

*Coloured is an official demographic category in South Africa for people of mixed ancestry

The mean age of caregivers (n=10) was 36 years (range: 29 – 48 years). Of the caregivers, eight were mothers and two were fathers. Six had completed Grade 12, two held a post-graduate diplomas/certificates, and three had completed college/university education. Seven caregivers were married, five were unemployed, and six had a monthly household income between R4,501 and R30,000 (equivalent to ∼ US$255 – 1702). Regarding perceived economic security, four felt they were ‘struggling’, two were ‘just getting by’, and four described themselves as ‘doing okay’ financially.

Nine of the 10 caregivers completed the technology questionnaire. Six caregivers owned android smartphones and two owned Apple smartphones. One caregiver did not report their phone type. All caregivers had personal email addresses. Eight accessed the internet primarily at home using either their own modem or mobile data. Caregivers rated their internet skills at an average of 8 on a 1-10 scale (1 indicates very poor skills and 10 indicates excellent skills).

### Acceptability, appropriateness and feasibility of the tele-delivered intervention

**Figure 2.**
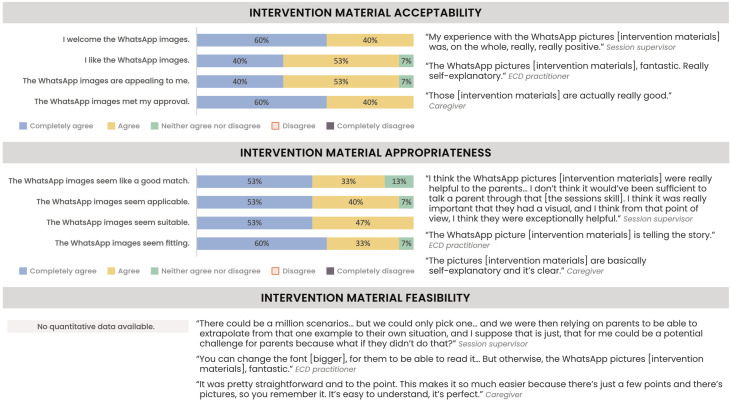
Intervention materials: Joint display of quantitative-qualitative results on acceptability, appropriateness and feasibility.

**Figure 3.**
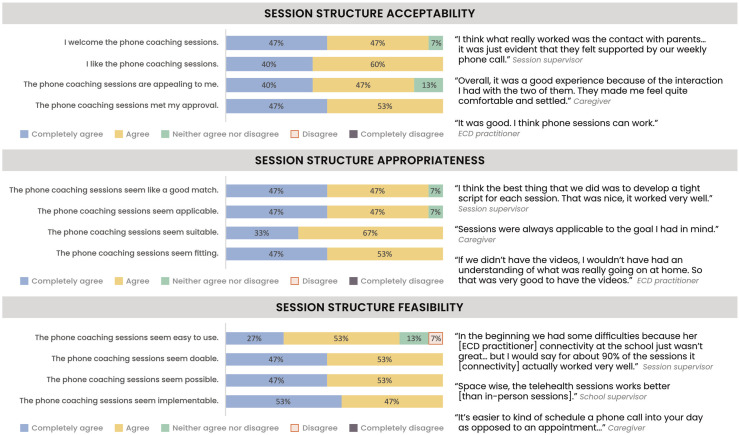
Session structure: Joint display of quantitative-qualitative results on acceptability, appropriateness and feasibility.

Quantitative survey and qualitative interview findigns were initially reviewed separately by participant group. As no meaningful differences in overall patterns or themes emerged between caregivers, ECD practitioners, and school and session supervisors, and given the small sample size, results were collapsed for reporting purposes. Results below are presented according to the three key technology components - (1) intervention materials, (2) provider training and supervision, and (3) and session structure. Figures 2 and 3 include joint displays for side-by-side comparison of qualitative and quantitative results relating to intervention materials and session structure. Barriers and facilitators are presented in table 3.

**Table 3.** Summary of implementation facilitators and barriers.

**Barriers**
The size of text on intervention materials was too small
Internet connectivity occasionally negatively affected session calls and video uploading
**Facilitators**
The use of images/pictures and minimal text in the intervention materials made them easy to use
ECD practitioners had protected time to attend training and supervision
Supportive caregiver-practitioner relationships
School leadership supported ECD practitioner attendance of training and supervision
“WhatsApp was a good choice”
Technical support (from research team) helped caregivers

### Intervention materials: WhatsApp images and accompanying text used to communicate intervention concepts

As indicated in Figure 2, quantitative and qualitative results showed similar findings about the acceptability and appropriateness of the intervention materials. Quantitative measures indicated that participants either completely agreed or agreed with 97% of statements about the acceptability of intervention materials (mean 4.5; range 3-5) and either completely agreed or agreed with 93% of appropriateness statements (mean 4.6; range 3-5). In addition to reporting that the intervention materials were acceptable, appropriate, and feasible, participants suggested ways to improve them. They recommended including more examples of caregivers using the strategies in everyday situations to support applying the skills in their own routines. One facilitator was identified - the minimal text used in the intervention materials which made them easy to use. Small text size was identified as a barrier and participants suggested that increasing the text size will improve intervention materials (see table 3).

### Provider training and supervision: Early childhood development practitioners and their school supervisors

Qualitative data were gathered from ECD practitioners, school supervisors and session supervisors, as these were the groups directly involved in training and supervision. ECD practitioners had prior training in the intervention content and training and supervision therefore focused specifically on session structure and technological aspects (see Figure 1).

ECD training and supervision was acceptable and described as “good”. Participants specifically mentioned that they found the mock sessions appealing, where ECD practitioners role-played intervention WhatsApp calls in preparation for real sessions. A session supervisor and ECD practitioner noted:

“I think what really worked there is the fact that we had those couple of weeks where the ECD practitioners weren’t working with families yet, and we used that time to provide supervision. So they had an hour of supervision a week, and during that supervision time, we basically roleplayed sessions, and we just worked our way through as many sessions as we could. That worked very well.” 

“In the beginning we concentrated a lot on trying to help me understand how to go about the questions [generalization], and how to ask the statements [self-reflection checklist], and how to assess the parent, I think that worked… That helped me a lot because it built my confidence and it helped me learn how to ask the questions to the parent, and how to address it if anything popped up… by the time I started my first session with a parent, my confidence was bold and I was feeling comfortable enough to start.” 

ECD practitioner training and supervision were appropriate and prepared practitioners adequately for sessions. Mock training sessions were again mentioned as particularly beneficial. Once sessions with caregivers started discussion of home-recorded videos during supervision was valuable because it provided an opportunity for ECD practitioners and supervisors to consider application of skills for each family based on their individual goals, needs, and progress. A session ECD practitioner and session supervisor commented:

“It worked if whoever is supervising me is also watching the caregiver-child video and we check in with each other before the session and run through what we see in the video, because maybe you see something that I didn’t notice.” 

“For me the most important thing is that she’s [ECD practitioner] watched the video before, I’ve watched the video before, and we’ve been able to talk about what we have both seen in that video, and what we would perhaps like the parent now to be able to just pay a bit more attention to.” 

Session supervisors and ECD practitioners reported that the training and supervision was feasible and could be successfully done in the school setting, provided they had protected time and support from school leadership. Support from principals and school supervisors for ECD training and supervision, as well as protected time were identified as an implementation facilitators (see table 3). Session supervisors noted:

“It really helps if the ECD worker has support from the school, and from the principal and from the supervisor in the school. And it really helps if they have allocated time that’s protected, so that it doesn’t feel like it’s just kind of squeezed into a very busy day. That there’s actually protected time for that [supervision], and that can be the ECD’s focus.” 

“What ended up happening is the ECD worker actually did the sessions from home, and I think that speaks to the support structure [at school]. But I think what it shows is that there is that flexibility to do these sessions from anywhere.” 

### Session structure: Tele-delivered caregiver coaching sessions

As shown in Figure 3, both quantitative and qualitative results suggested that participants perceived the session structure comprising of an asynchronous component (recording and uploading a 5-minute caregiver–child interaction video) and synchronous component (a session conducted via a WhatsApp call) as acceptable, appropriate, and feasible. Quantitative results indicated that participants either completely agreed or agreed with 95% of acceptability statements (mean 4.4; range 3-5); either completely agreed or agreed with 97% of appropriateness statements (mean 4.5; range 3-5); and either completely agreed or agreed with 95% of feasibility statements (mean 4.4; range 2-5).

The qualitative data showed that while participants found the session structure acceptable, appropriate, and feasible (Figure 3), they also mentioned some aspects that were challenging at first. Thinking out loud during the generalization section on how they would apply session skills in their daily lives was a new, unfamiliar experience for some caregivers and took some getting used to. However, they found this part of the sessions valuable and described it as “a particularly good thing”. While home-recorded videos were considered appropriate, caregivers would have liked more detailed feedback on their videos and suggested recording longer videos to better show their child’s progress and allow for more comprehensive feedback on their skill application. Session supervisors also recommended that the purpose of videos should be explained more clearly at the start. The process of recording and uploading videos required technical skills and some caregivers initially struggled with tasks like positioning the camera, capturing their child’s actions during a continuous 5-minute long video recording and uploading videos.

Participants described WhatsApp as “a good choice” for delivering the intervention, highlighting it as an important technological facilitator (see table 3). Caregivers also felt well supported by ECD practitioners, explaining that their interactions made sessions “a good experience” and helped overcome initial concerns that online sessions might feel impersonal or awkward (see table 3). Technical support from the research team was another technological facilitator that further enabled participation, as it “really helped” when caregivers had trouble uploading videos or connecting to calls. Coverage of data costs was also mentioned as facilitator. Internet connectivity was identified as an implementation barrier (see table 3), but it was noted that although occasional connectivity problems impacted video uploading and session calls, participants were able to work around these issues and could successfully use the necessary technology.

## Discussion

This study provides pilot (proof-of-concept) evidence for the acceptability, appropriateness and feasibility of a tele-delivered caregiver coaching intervention in a low-resource South African context and on implementation barriers and facilitators to the tele-delivery. Both qualitative and quantitative results suggested that the three key technological components – intervention materials, provider training and supervision and session structure – were overall perceived as acceptable, appropriate and feasible by participating session supervisors, ECD practitioners, school supervisors and caregivers. Facilitators that supported implementation included using a low-cost delivery platform (WhatsApp), minimal text in intervention materials used to communicate intervention concepts, protected time for ECD practitioners, support from school leadership, the supportive caregiver-practitioner relationship, remote technical support from team members and coverage of data costs. Barriers were the small text size on intervention materials and poor internet connectivity.

The first key technological component, intervention materials, was specifically designed for easy sharing and viewing via WhatsApp, using simple visuals, minimal text and small file sizes that to reduce mobile data usage. The use of minimal text and visual aids has been shown in the literature to be an accepted and effective way for digitally communicating health concepts to persons with low literacy levels.^57^ This aligns with participant feedback which reported that the intervention materials (images and simple text shared via WhatsApp) “told the story” and were both easy to understand and access on their phones. However, our findings indicate that the intervention materials were best suited for use with provider contact, rather than as a standalone resource. Weekly WhatsApp calls played a critical role in helping caregivers connect the examples in the intervention materials to their own specific situations, increasing the likelihood of them understanding and applying intervention skills. These findings align with prior research emphasizing that tele-delivered autism interventions are most effective when digital tools/resources are combined with ongoing provider engagement. Interventions that incorporate synchronous (real-time) coaching or hybrid approaches (where caregivers review content asynchronously but still receive real-time provider support) have been shown to be more effective and produce better intervention outcomes (such as increased caregiver engagement and improved child development) compared to fully asynchronous or self-directed online learning formats.^27,29,58–60^

The second key technological component, provider training and supervision, was considered acceptable, appropriate, and feasible as long as there were supports from school leadership and protected time for training and supervision activities. This finding aligns with other studies on the challenges of delivering autism interventions, which emphasize that successful digital health interventions need more than just the right technology – they also need to be part of a supportive system with the right structures in place.^61^ The mock sessions were especially helpful, as they offered a low-pressure space to practice skills, receive feedback and build confidence before working with families. Conducting these sessions over WhatsApp also allowed practitioners to become familiar with the technology, the coaching process, and intervention content while closely simulating real session conditions.

The third key technological component we evaluated was the session structure. Using WhatsApp, a low-cost and widely used messaging platform, familiar to both caregivers and ECD practitioners, was a key technological facilitator. WhatsApp’s low data usage and ability to work on slow internet connections may have improved accessibility and highlights the importance of technological fit: choosing tools participants already use can make digital health interventions more accessible and sustainable.^62–64^ Caregivers reported that the relationships they formed with ECD practitioners and supervisors during the session was a facilitator that made the tele-delivered coaching sessions acceptable, comfortable and personal. This positive experience aligns with literature that that suggests a collaborative coaching approach and strong caregiver-provider relationships improves coaching acceptability and help caregivers learn skills.^65–68^ Scheduling challenges and time constraints have been reported as a challenge to in-person coaching^69^ and it was therefore promising that caregivers appreciated the flexibility of tele-delivered sessions, noting that they were easier to schedule than in-person visits since there was no need to travel to schools or clinics. The asynchronous element may have provided additional flexibility by allowing caregivers to complete some activities at times that suited them best. They also found it helpful that children were not present during these sessions, which reduced logistical challenges.

Despite the reported fit between the session structure and context, poor internet connectivity was a technological barrier that occasionally disrupted video uploads and WhatsApp calls. This reflects a common tension in digital health - while technology can increase access and reduce logistical burdens, it can also introduce new barriers.^27,70^ Ensuring participants knew that the research team was available to help them problem-solve if they were unable to upload their videos or connect to a call was instrumental in overcoming this barrier (internet connectivity) and maintaining caregiver engagement. This finding is consistent with previous research highlighting the importance of accessible technology assistance in facilitating tele-delivery and being a predictor of participant engagement.^60,71,72^

These findings may have implications for scalability beyond the South African context. The intervention was intentionally designed for resource-constrained settings, and its use of low-cost, widely available technology, simple intervention materials, and a task-sharing model may support use in similar low-resource settings beyond South Africa. However, for scale-up beyond a research context, factors that facilitated implementation, such as technical support, coverage of data costs, and non-specialist training and supervision, would need to be embedded within existing public sector or community-based systems, such as health, education, or early childhood services. This may require dedicated personnel, protected time, and partnerships with telecommunications providers to support implementation and improved data access for families.

These findings may also be relevant beyond LMIC settings. Challenges such as limited workforce capacity, long waiting times, and high costs are increasingly evident in high-income contexts.^73,74^ Approaches developed in low-resource settings, such as task-sharing models that rely on minimal internet bandwidth and simple technology, may offer more affordable and flexible options for broader implementation. However, these findings should be interpreted as preliminary and require further investigation in larger and more diverse samples.

In addition, ongoing challenges related to the digital divide highlight the need for policy that supports the implementation of digital delivery models that can work with unstable internet and low data access. This includes approaches that can function when connectivity is interrupted and that combine asynchronous and synchronous elements to reduce the need for continuous internet access. Partnerships with telecommunications providers may help improve data access and connectivity for families, for example through zero-rated platforms, subsidised data bundles, or programme-specific data support. These strategies may help extend reach and maintain engagement in resource-constrained settings, while recognising that internet infrastructure and affordability remain outside the direct control of intervention developers. These approaches also align with the WHO resolution on autism, which calls for strengthened infrastructure, greater use of technology, and improved support for families.^1^ These implications should be considered preliminary given the pilot nature of the study.

This is, to our knowledge, one of the first studies in a LMIC to evaluate a hybrid (synchronous-asynchronous) tele-delivered autism caregiver coaching intervention delivered via a low-cost instant messaging platform (WhatsApp). While similar studies relied on video conferencing platforms, our use of WhatsApp may offer a more accessible approach for LMIC, although further research is needed to evaluate its scalability. Overall, our findings support previous research indicating that tele-delivered autism caregiver coaching is acceptable, appropriate, and feasible.^21,27,30,75^ Furthermore, results indicate that this intervention fits well with the South African context it was designed for, which may increase its potential to be impactful and sustainable, although further evaluation in larger and more diverse samples is needed.^41,76^

### Study limitations

First, most participating caregivers had smartphones and relatively high self-rated internet skills, which may mean that participants in the study were those families who had access to technology and were comfortable using it, while families facing greater technological barriers were less likely to participate. The views expressed by participants in this study may therefore not fully reflect those of families facing greater technological barriers or the broader population of South African caregivers. However, efforts were made to include and support participants who were hesitant about technology. For example, the research team provided remote assistance during sessions and video uploads, and participants were given the option to conduct their sessions via WhatsApp video or voice calls. Although caregivers were required to record and upload a weekly caregiver-child interaction video, they could use any phone or device to do so. Secondly, we acknowledge that the project coordinator, who was also the session supervisor for some dyads and familiar to all participants, conducted the qualitative interviews, which could have influenced participants’ responses. However, this existing relationship may also have made caregivers feel more comfortable and open during the interviews, and therefore more likely to share their viewpoints, both positive and negative, freely. Lastly, we acknowledge that the sample size was small, and the findings may not be generalizable to other caregivers of young autistic children in South Africa or more broadly across low-resource settings. However, this study examined implementation outcomes of hybrid (synchronous-asynchronous) telehealth approached delivered via a low-cost platform (WhatsApp) in a LMIC context, and an important step towards better understanding what makes tele-delivered interventions work in these settings.

## Conclusion

There is a growing global interest in using technology to support autism service delivery. To our knowledge, this is one of the first studies to evaluate key implementation outcomes of a hybrid caregiver coaching intervention combining synchronous and asynchronous delivery using a low-cost platform (WhatsApp). Caregivers and practitioners involved in the sessions found the intervention acceptable, appropriate, and feasible, highlighting factors that they liked, that matched their needs and that made the intervention easy to use. They also identified several challenges and areas of improvement. These findings offer valuable insights into the factors that contribute to the success of tele-delivered interventions and point to areas that may require adaptation in future implementations. A key next step is to assess whether the tele-delivered intervention lead to changes in caregiver and child behaviours and whether home-recorded videos, made with low-cost smartphones, can be used to measure intervention outcomes. Another important next step is to examine coaching fidelity as an implementation outcome.

## Supplemental material

Supplemental material - Tele-delivered caregiver coaching for autism in South Africa – A mixed-methods study of acceptability, appropriateness and feasibility Supplemental material for Tele-delivered caregiver coaching for autism in South Africa – A mixed-methods study of acceptability, appropriateness and feasibility by Marisa Viljoen, Zahrah Ismail Dawood, Noleen Seris, Nokuthula Shabalala, Minkateko Ndlovu, Petrus J de Vries and Lauren Franz in Digital Health.

Supplemental material - Tele-delivered caregiver coaching for autism in South Africa – A mixed-methods study of acceptability, appropriateness and feasibility Supplemental material for Tele-delivered caregiver coaching for autism in South Africa – A mixed-methods study of acceptability, appropriateness and feasibility by Marisa Viljoen, Zahrah Ismail Dawood, Noleen Seris, Nokuthula Shabalala, Minkateko Ndlovu, Petrus J de Vries and Lauren Franz in Digital Health.

Supplemental material - Tele-delivered caregiver coaching for autism in South Africa – A mixed-methods study of acceptability, appropriateness and feasibility Supplemental material for Tele-delivered caregiver coaching for autism in South Africa – A mixed-methods study of acceptability, appropriateness and feasibility by Marisa Viljoen, Zahrah Ismail Dawood, Noleen Seris, Nokuthula Shabalala, Minkateko Ndlovu, Petrus J de Vries and Lauren Franz in Digital Health.

Supplemental material - Tele-delivered caregiver coaching for autism in South Africa – A mixed-methods study of acceptability, appropriateness and feasibility Supplemental material for Tele-delivered caregiver coaching for autism in South Africa – A mixed-methods study of acceptability, appropriateness and feasibility by Marisa Viljoen, Zahrah Ismail Dawood, Noleen Seris, Nokuthula Shabalala, Minkateko Ndlovu, Petrus J de Vries and Lauren Franz in Digital Health.

## Data Availability

The datasets generated during and/or analysed during the current study are available from the corresponding author on reasonable request.*
