# Supplementary material for: Tele-delivered caregiver coaching for autism in South Africa – A mixed-methods study of acceptability, appropriateness and feasibility
Source: Digit Health. 2026 Jun 11;12:20552076261459555. doi: 10.1177/20552076261459555 (PMC13261048; doi:10.1177/20552076261459555)
Supplement: Supplemental material - Tele-delivered caregiver coaching for autism in South Africa – A mixed-methods study of acceptability, appropriateness and feasibility [file sj-pdf-1-dhj-10.1177_20552076261459555.pdf]

Summary of quantitative results

	**Caregivers (n = 9)**	**ECD practitioners & school supervisors (n = 3)**	**Session supervisors (n = 3)**	**All participants (n = 15)**
	Mean (range)	Mean (range)	Mean (range)	Mean (range)
Acceptability – intervention materials (AIM)	4.3 (3 – 5)	4.7 (4 – 5)	4.9 (4 – 5)	4.5 (3 – 5)
Appropriateness - intervention materials (IAM)	4.4 (3 – 5)	4.7 (4 – 5)	5 (5)	4.6 (3 – 5)
Acceptability – session structure (AIM)	4.2 (3 – 5)	4.7 (4 – 5)	4.8 (4 – 5)	4.4 (3 – 5)
Appropriateness – session structure (IAM)	4.5 (3 – 5)	4.7 (4 – 5)	4.5 (4 – 5)	4.5 (3 – 5)
Feasibility – session structure (FIM)	4.1 (2 – 5)	4.7 (4 – 5)	4.9 (4 – 5)	4.4 (2 – 5)
