# Supplementary material for: Tele-delivered caregiver coaching for autism in South Africa – A mixed-methods study of acceptability, appropriateness and feasibility
Source: Digit Health. 2026 Jun 11;12:20552076261459555. doi: 10.1177/20552076261459555 (PMC13261048; doi:10.1177/20552076261459555)
Supplement: Supplemental material - Tele-delivered caregiver coaching for autism in South Africa – A mixed-methods study of acceptability, appropriateness and feasibility [file sj-pdf-2-dhj-10.1177_20552076261459555.pdf]

Tele-delivered caregiver coaching for autism in South Africa – a mixed-methods study of acceptability, appropriateness and feasibility: supplementary material – summary of qualitative results

Theme	Acceptability	Appropriateness	Feasibility	Facilitators	Barriers
**Intervention materials (WhatsApp images and accompanying text)**	Intervention materials were acceptable<br><br>BUT<br>Some caregivers would have liked more examples of how skills can be applied in everyday activities.	Intervention materials were appropriate – communicated intervention concepts well and accurately.<br><br>BUT<br>one caregiver reported the toys in intervention materials (WhatsApp pictures) was not available to them	Intervention materials were feasible – for both session and home use.<br><br>BUT<br>Caregivers struggled to generalize skills to their situations which were not identical to those in the images – more examples would help with this (also see acceptability).	Minimal text = images easy to use.	Small text size in the WhatsApp images were difficult to see.
**Session structure**	Session structure was acceptable<br><br>Home-recorded caregiver-child interaction videos were acceptable and easy to record. Some participants enjoyed making them<br><br>BUT,<br>Some initially struggled and found it stressful to record a continuous 5-minute long video.	Session structure was appropriate - sessions were applicable to the goal caregivers had in mind.<br><br>Some reported that home-recorded caregiver-child interaction videos were often too short to accurately show child skills/change. Some caregivers wanted the option to record longer videos.	Session structure was feasible – convenient and easy to fit into day for most.<br><br>Recording (positioning camera so that it captures caregiver and child actions) & uploading home-recorded caregiver-child interaction videos were initially difficult for some.	WhatsApp was a good intervention delivery platform choice.<br><br>Support from ECD practitioners and/or session supervisors. Caregivers liked the collaborative coaching approach.<br><br>Technical support from research team during video recording and uploading, or session call.	Internet connectivity was a challenge (barrier) at times – exacerbated by loadshedding. Caregivers and ECD practitioners could work around connectivity issues.

		Session supervisors felt the purpose of videos were unclear to some caregivers. Suggested clearer explanation of purpose.<br><br>Answering questions during generalization section of sessions was difficult & repetitive for some caregivers. Others described the generalization section as particularly useful.<br><br>Some wanted more direct feedback on their use of skills in their weekly home-recorded caregiver-child interaction videos.		Data costs covered by study (for caregivers and ECD practitioners).	
**ECD practitioner training and supervision**	ECD practitioner training and supervision was acceptable. Participants liked the mock sessions.	ECD practitioner training and supervision was appropriate – Supervision was appropriate and prepared ECD practitioners for	ECD practitioner training and supervision feasible – on the condition that ECD practitioners had protected time for sessions and support from school	ECD practitioner has protected time and support from school leadership for training and supervision.	

		sessions. Mock training sessions = particularly beneficial.<br><br>Supervision: discussion of home-recorded videos provided opportunity to talk about each dyad’s goals, needs, and progress.	leadership/principals.		
