# Supplementary material for: Tele-delivered caregiver coaching for autism in South Africa – A mixed-methods study of acceptability, appropriateness and feasibility
Source: Digit Health. 2026 Jun 11;12:20552076261459555. doi: 10.1177/20552076261459555 (PMC13261048; doi:10.1177/20552076261459555)
Supplement: Supplemental material - Tele-delivered caregiver coaching for autism in South Africa – A mixed-methods study of acceptability, appropriateness and feasibility [file sj-pdf-3-dhj-10.1177_20552076261459555.pdf]

Therapist Teacher Demographics

____

DEMOGRAPHICS

1. Gender
   - ◯ Female
   - ◯ Male
   - ◯ Refused
   - ◯ Unknown

2. Level of education
   - ◯ Tertiary
   - ◯ Certificate
   - ◯ Grade 12/matric
   - ◯ Grades 9-11
   - ◯ Grade 8 and below
   - ◯ No formal education
   - ◯ Unknown

3. Ethnicity
   - ◯ African
   - ◯ Colored
   - ◯ Indian
   - ◯ White
   - ◯ Other

3a. Please specify ________________________________

4. Position
   - ◯ ESDM therapist
   - ◯ School supervisor
   - ◯ Early childhood development worker
   - ◯ Other

4a. Please specify ________________________________

5. First language
   - ◯ isiXhosa
   - ◯ Afrikaans
   - ◯ English
   - ◯ isiZulu
   - ◯ Other

5a. Please specify ________________________________

6. What other languages do you speak? ________________________________

Date of form completion ________________________________

Demographics

DEMOGRAPHICS

Child gender	◯ Female<br>◯ Male<br>◯ Refused
Child first name	______________________
Child last name	______________________
Child middle name	______________________
Child date of birth	______________________
Child city of birth	______________________
Child age at study entry	______________________<br>(Months)
Child ethnicity	◯ African<br>◯ Colored<br>◯ Indian<br>◯ White<br>◯ Other
Please specify	______________________
Child home language	◯ isiXhosa<br>◯ Afrikaans<br>◯ English<br>◯ isiZulu<br>◯ Other
Please specify	______________________
Child age of first concern for autism	______________________<br>(Months)
Child age at autism diagnosis	______________________<br>(Months)
How many hours of schooling, intervention and/or therapy does child receive per week	______________________<br>(Total hours per week)

Primary caregiver enrolled in study	◯ Mother<br>◯ Father<br>◯ Grandmother<br>◯ Grandfather<br>◯ Aunt<br>◯ Uncle<br>◯ Institution<br>◯ Other family member
Please specify	__________________________________
Caregiver gender	◯ Female<br>◯ Male<br>◯ Refused
Primary caregiver first name	__________________________________
Primary caregiver last name	__________________________________
Primary caregiver middle name	__________________________________
Primary caregiver date of birth	__________________________________
Primary caregiver city of birth	__________________________________
Primary caregiver age	__________________________________<br>(Years)
Primary caregiver ethnicity	◯ African<br>◯ Colored<br>◯ Indian<br>◯ White<br>◯ Other
Please specify	__________________________________
Primary caregiver marital status	◯ Married<br>◯ Live-in partner<br>◯ Partner not live-in<br>◯ Single
Primary caregiver level of education	◯ Tertiary<br>◯ Post-grade 12 certificate or diploma<br>◯ Grade 12/ Matric<br>◯ Grade 9-11<br>◯ Grade 8 and below<br>◯ No formal educaton<br>◯ Unknown
Please specify	__________________________________

Primary caregiver occupation	◯ Not currently working<br>◯ Employed, part-time/casual<br>◯ Employed full-time
What is your family's total household income per month?	◯ Less than R4500 per month<br>◯ Between R4 501 and R12 500 per month<br>◯ Between R12 501 and R30 000 per month<br>◯ Between R30 001 and R52 000 per month<br>◯ Between R52 001 and R70 000 per month<br>◯ More than R70 001 per month
When you think of the total income of your family, do you consider your family to be	◯ Struggling<br>◯ Just getting by<br>◯ Doing okay<br>◯ Managing well with some extra money left over at the end of the month<br>◯ Well off
Type of household dwelling	◯ Temporary self-built<br>◯ Permanent self-built<br>◯ Government built<br>◯ Professionally built flat<br>◯ Professionally built house
Type of household toilet	◯ Flush toilet inside home<br>◯ Flush toilet outside home<br>◯ Bucket<br>◯ Outside pit<br>◯ Chemical<br>◯ None
Rely on public transportation	◯ Yes ◯ No
Number of children living in the home with child (not including child in study)	______________________________
Number of adults living in the home with child	______________________________
Date of form completion	______________________________

Acceptability of Intervention Measure (AIM):

	Completely disagree	Disagree	Neither agree nor disagree	Agree	Completely agree
1. The phone coaching sessions met my approval.	①	②	③	④	⑤
2. The phone coaching sessions are appealing to me.	①	②	③	④	⑤
3. I like the phone coaching sessions.	①	②	③	④	⑤
4. I welcome the phone coaching sessions.	①	②	③	④	⑤

Acceptability of Intervention Measure (AIM): WhatsApp messages

	Completely disagree	Disagree	Neither agree nor disagree	Agree	Completely agree
1. The WhatsApp messages met my approval.	①	②	③	④	⑤
2. The WhatsApp messages are appealing to me.	①	②	③	④	⑤
3. I like the WhatsApp messages.	①	②	③	④	⑤
4. I welcome the WhatsApp messages.	①	②	③	④	⑤

Intervention Appropriateness Measure (IAM): Telephone Coaching

	Completely disagree	Disagree	Neither agree nor disagree	Agree	Completely agree
1. The phone coaching sessions seem fitting.	①	②	③	④	⑤
2. The phone coaching sessions seem suitable.	①	②	③	④	⑤
3. The phone coaching sessions seem applicable.	①	②	③	④	⑤
4. The phone coaching sessions seem like a good match.	①	②	③	④	⑤

20b. Intervention Appropriateness Measure (IAM): WhatsApp messages

	Completely disagree	Disagree	Neither agree nor disagree	Agree	Completely agree
1. The WhatsApp messages seem fitting.	①	②	③	④	⑤
2. The WhatsApp messages seem suitable.	①	②	③	④	⑤
3. The WhatsApp messages seem applicable.	①	②	③	④	⑤
4. The WhatsApp messages seem like a good match.	①	②	③	④	⑤

Feasibility of Intervention Measure (FIM): Telephone Coaching

	Completely disagree	Disagree	Neither agree nor disagree	Agree	Completely agree
1. The phone coaching sessions seem implementable.	①	②	③	④	⑤
2. The phone coaching sessions seem possible.	①	②	③	④	⑤
3. The phone coaching sessions seem doable.	①	②	③	④	⑤
4. The phone coaching sessions seem easy to use.	①	②	③	④	⑤

Technology Questionnaire

Technology Use

Subject ID ________________________

Date of Form Completion ________________________

1. Do you own a cell phone?	○ Yes	○ No	○ NA
a. If yes, is it a smart phone? (e.g. Internet, apps, videos)	○ Yes	○ No	○ NA
b. If yes, is it a standard phone? (e.g. voice and SMS, no Internet or video)	○ Yes	○ No	○ NA
c. If yes, what make is your phone (e.g Samsung, Huawei, Nokia)	_________________________		
2. Do you have a personal email address?	○ Yes	○ No	○ NA
3. Do you use social media?	○ Yes	○ No	○ NA
a. Facebook	○ Yes	○ No	○ NA
b. WhatsApp	○ Yes	○ No	○ NA
c. Twitter	○ Yes	○ No	○ NA
d. Instagram	○ Yes	○ No	○ NA
e. Other: ____________________			
4. Do you connect to the Internet	○ Yes	○ No	○ NA
a. If so, where?			
Community	○ Yes	○ No	○ NA
Work	○ Yes	○ No	○ NA
Home	○ Yes	○ No	○ NA
5. If you connect at home, which of the following applies?			
a. We have our own modem and internet service	○ Yes	○ No	○ NA
b. We connect via free community Wi-Fi	○ Yes	○ No	○ NA
c. We use cell phone data	○ Yes	○ No	○ NA

6. On a scale of 1 (very poor) to 10 (excellent), rate your own skill with using the Internet, and Videos): ________________
