# Supplementary material for: Tele-delivered caregiver coaching for autism in South Africa – A mixed-methods study of acceptability, appropriateness and feasibility
Source: Digit Health. 2026 Jun 11;12:20552076261459555. doi: 10.1177/20552076261459555 (PMC13261048; doi:10.1177/20552076261459555)
Supplement: Supplemental material - Tele-delivered caregiver coaching for autism in South Africa – A mixed-methods study of acceptability, appropriateness and feasibility [file sj-pdf-4-dhj-10.1177_20552076261459555.pdf]

ASSESS checklist - Tele-delivered caregiver coaching for autism in South Africa – a mixed-methods study of acceptability, appropriateness and feasibility

REVIEW OR META-ANALYSIS QUESTION	
**Item**	**Reported on page #**
The overall question guiding the review or meta-analysis	n/a
**ARTICLE CITATION**	
**Item**	**Reported on page #**
Study author, publication year	p. 1
Study title	p. 1
**INTRODUCTION - IMPLEMENTATION AND INTERVENTION STRATEGY**	
**Item**	**Reported on page #**
Rationale	p. 3-4
Aim(s), objective(s), or research question(s)	p. 5
**METHODS: DESCRIPTION**	
**Item**	**Reported on page #**
Descriptions	p. 4-5
Adaptation	p. 4-5
Design	p. 5
Participant types	p. 5-6
Comparison group	n/a
Context	p. 3-4
Sites	p. 6
Subgroups (optional)	n/a
Implementation phase	p. 6
Process evaluation	p. 5
Sample size	p. 5-6
Analysis	p. 7-8
Sub-group analyses	n/a
**METHODS: DESCRIPTION - IMPLEMENTATION OUTCOMES**	
**Item**	**Reported on page #**
Outcomes (implementation)	p. 4; 7
Acceptability	p. 4; 7
Adoption	n/a
Appropriateness	p. 4; 7
Feasibility	p. 4; 7
Fidelity	n/a
Cost	n/a
Penetration	n/a
Sustainability	n/a
**METHODS: DESCRIPTION INTERVENTION OUTCOMES**	
**Item**	**Reported on page #**
Outcomes (intervention)	n/a
**RESULTS: DESCRIPTION – IMPLEMENTATION OUTCOMES**	
**Item**	**Reported on page #**

Outcomes (implementation findings)	p. 11-13
Acceptability	p. 11-13
Adoption	n/a
Appropriateness	p. 11-13
Feasibility	p. 11-13
Fidelity	n/a
Cost	n/a
Penetration	n/a
Sustainability	n/a
**RESULTS: DESCRIPTION – INTERVENTION OUTCOMES**	
**Item**	**Reported on page #**
Outcomes (intervention findings)	n/a
**ADDITIONAL FINDINGS**	
**Item**	**Reported on page #**
Barriers to implementation	p. 11; 13
Facilitators of implementation	p. 11-13
**METHODS: EVALUATION**	
**Item**	**Reported on page #**
Design (mixed methods)	p. 5
Mixed methods criteria	
Is there an adequate rationale for using a mixed methods design to address the research question?	Yes. p. 5; 8
Are the different components of the study effectively integrated to answer the research question?	Yes. p. 8; p. 11; p. 13
Are the outputs of the integration of qualitative and quantitative components adequately interpreted?	Yes. p. 13-15
Are divergences and inconsistencies between quantitative and qualitative results adequately addressed?	Yes. p. 13-15
Do the different components of the study adhere to the quality criteria of each tradition of the methods involved?	Yes. p. 7-8
**RESULTS: EVALUATION**	
**Item**	**Reported on page #**
Bias / evaluation	n/a
